# Enhanced Sensitivity of Gas Sensor Based on Poly(3-hexylthiophene) Thin-Film Transistors for Disease Diagnosis and Environment Monitoring

**DOI:** 10.3390/s150409592

**Published:** 2015-04-22

**Authors:** Marco R. Cavallari, José E. E. Izquierdo, Guilherme S. Braga, Ely A. T. Dirani, Marcelo A. Pereira-da-Silva, Estrella F. G. Rodríguez, Fernando J. Fonseca

**Affiliations:** 1 Departamento de Engenharia de Sistemas Eletrônicos, Escola Politécnica da Universidade de São Paulo (EPUSP), Av. Prof. Luciano Gualberto, trav. 3, n. 158, Cidade Universitária, CEP 05508-900, São Paulo-SP, Brasil; E-Mails: rcavallari@lme.usp.br (M.R.C.); jeeizquierdo@lme.usp.br (J.E.E.I.); gbraga@lme.usp.br (G.S.B.); dirani@pucsp.br (E.A.T.D.); 2 Instituto Superior Politécnico José Antonio Echeverría (ISPJAE), Centro de Investigaciones de Microelectrónica (CIME), Antigua Carretera de Vento, km 8 1/2, Boyeros, CP 10800 La Habana, Cuba; E-Mail: estrella@electrica.cujae.edu.cu; 3 EMBRAPA Instrumentação, Rua Quinze de Novembro, 1452-Centro, CEP 13560-970 São Carlos, SP, Brasil; 4 Pontifícia Universidade Católica de São Paulo (PUC-SP), Rua Marquês de Paranaguá, 111-Consolação, CEP 01303-050 São Paulo-SP, Brasil; 5 Instituto de Física de São Carlos–USP, Av. Trabalhador Sãocarlense 400, CEP 13566-590 São Carlos-SP, Brasil; E-Mail: maps@ifsc.usp.br; 6 Centro Universitário Central Paulista–UNICEP, Rua Miguel Petroni 5111, CEP 13563-470 São Carlos-SP, Brasil

**Keywords:** organic thin-film transistors, gas sensors, P3HT, volatile organic compounds

## Abstract

Electronic devices based on organic thin-film transistors (OTFT) have the potential to supply the demand for portable and low-cost gadgets, mainly as sensors for *in situ* disease diagnosis and environment monitoring. For that reason, poly(3-hexylthiophene) (P3HT) as the active layer in the widely-used bottom-gate/bottom-contact OTFT structure was deposited over highly-doped silicon substrates covered with thermally-grown oxide to detect vapor-phase compounds. A ten-fold organochloride and ammonia sensitivity compared to bare sensors corroborated the application of this semiconducting polymer in sensors. Furthermore, P3HT TFTs presented approximately three-order higher normalized sensitivity than any chemical sensor addressed herein. The results demonstrate that while TFTs respond linearly at the lowest concentration values herein, chemical sensors present such an operating regime mostly above 2000 ppm. Simultaneous alteration of charge carrier mobility and threshold voltage is responsible for pushing the detection limit down to units of ppm of ammonia, as well as tens of ppm of alcohol or ketones. Nevertheless, P3HT transistors and chemical sensors could compose an electronic nose operated at room temperature for a wide range concentration evaluation (1–10,000 ppm) of gaseous analytes. Targeted analytes include not only biomarkers for diseases, such as uremia, cirrhosis, lung cancer and diabetes, but also gases for environment monitoring in food, cosmetic and microelectronics industries.

## 1. Introduction

Nowadays, consumers demand portable and low-cost electronic devices, mainly as sensors for *in situ* medical diagnosis and the agribusiness industry. One of the most important applications is in monitoring and detecting volatile organic compounds (VOCs), including isoprene, acetone, ethanol and methanol, which are exhaled during respiration as a result of various metabolic processes [1]. Acetone detection is considered one of the main important targets of the clinical analysis market, as it can provide useful information to diagnose diabetes or other glucose-related dysregulation [2]. Hyperglycemic patients present an increased concentration of ketone bodies, not only in the blood, but also in exhaled breath from 100 ppb to 2 ppm or more [3,4]. In diabetic patients, glucose level dysregulation and, consequently, its metabolism are due to the deficiency or even absence of cellular insulin receptor expression. Furthermore, this ketone biomarker can also be present in exhaled air from patients with lung cancer [5]. Another important biomarker in exhaled breath is ammonia, which can be associated with *Helicobacter pylori* colonies in the gastrointestinal tract [2]. This notwithstanding, its concentration lies within 1 ppm, a range associated with other infirmities, such as uremia or renal failure (0.278 < *c* < 4.8 ppm) [6] and hepatic cirrhosis (0.278 < *c* < 0.745 ppm) [7]. Current diagnosis methods are invasive and demand body piercing or cutting procedures. Additionally, blood analysis to assess glucose level requires needles and reactive tapes, which cost and generate trash with potential biological risk. Consequently, further specialized services of collection and incineration are necessary to deal with these infecting residues.

Ammonia detection can find application for environment monitoring in the food industry. For instance, it must be kept below 25 ppm in chicken farms to prevent respiratory diseases and secondary infections [8]. On the other hand, chlorinated solvents are widely employed to process semiconducting polythiophenes in research laboratories and to perform conventional silicon photolithography [9,10,11,12,13,14,15]. According to studies from the nineties, organochloride compounds, such as chloroform and chlorobenzene, are associated with an increase of cancer incidence in the electronic industry [16], mainly breast cancer [14,15]. It is also believed that an increased mortality rate by cancer might be associated with descendants of these employees [15].

Among organic materials in the present, P3HT is a widely-used p-type semiconductor for solar cells [17] and transistors [18,19]. Organic thin-film transistors (OTFTs) have been considered a potential engineering solution for gas [9] and liquid analyte detection [10] in recent years, largely due to their low manufacturing cost at temperatures well below 200 °C. A well-known OTFT structure in sensing is the bottom-gate/bottom-contact field-effect transistor (FET) over highly-doped silicon substrates and inorganic dielectrics [9,11,12,13]. Similarly to chemical sensors, a polymeric semiconductor placed on top of an insulator and amidst the electrodes acts usually as the sensing layer. Nevertheless, in this case, analyte interaction is mostly confined to a nanometric region with increased charge concentration, called the transistor channel [20,21]. In this context, this paper demonstrates the potential of gas sensors based on P3HT TFTs and chemical sensors for non-invasive disease diagnosis and environment monitoring through the detection of gaseous analytes, such as ammonia, ketones and organochlorides.

## 2. Experimental Section 

### 2.1. Sensors Preparation

**Figure 1:**
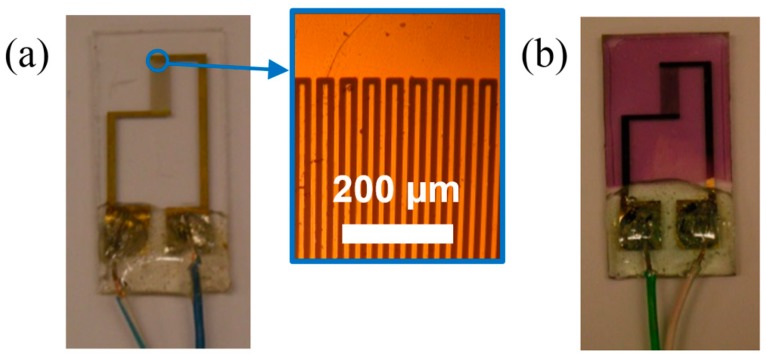
Chemical sensors in this work: (**a**) bare sensor; (**b**) poly(3-hexylthiophene) (P3HT) film-based sensor. Inset: optical micrograph of the interdigitated electrodes.

The non-specific chemical sensors in Figure 1 were processed over BK7 glass substrates (Opto Eletrônica S/A, São Carlos, Brazil) of 10 mm × 25 mm × 1 mm. Initial cleaning consisted of 10-min ultrasonic baths of neutral liquid detergent (Extran^®^ MA 02, Merck Millipore, Darmstadt, Germany), acetone (ACS reagent, Sigma-Aldrich, MO, USA) and isopropyl alcohol (ACS reagent, Sigma-Aldrich, MO, USA), with an intermediary rinse in ultra-pure water and a final blow dry under nitrogen. Interdigitated electrodes in Figure 1a were defined by chemically etching nichrome (Ni–Cr 80/20 wt ratio) (10 nm) and gold (100 nm) films deposited by direct current (DC) sputtering and patterned by conventional micrometric photolithography. Microelectrodes were composed of 50 digits (10 μm wide and 5 mm long) with an interdigital spacing of 10 μm (more details in Braga *et al.* [22]). P3HT (regioregularity >95%, *M_w_* = 10–35 kDa, *M_w_*/*M_n_* = 2; ADS, Quebec, Canada) sensors, as in Figure 1b, were obtained from 7 mg/mL P3HT solution in toluene spun at 600 and 3000 rpm for 30 s to form a 79 ± 4 and 44 ± 3 nm-thick film, respectively. Toluene was chosen not only for its higher boiling point when compared to chloroform, but also because its chemical structure, C_7_H_8_, does not contain any chlorine atoms. Due to low solubility, all P3HT solutions herein were agitated for 24 h at room temperature and heated on a hot plate for 3 h at 60 °C prior to filtration at 0.45 µm. Thermal annealing was performed at 140 °C during 15 min on a hot plate.

**Figure 2:**
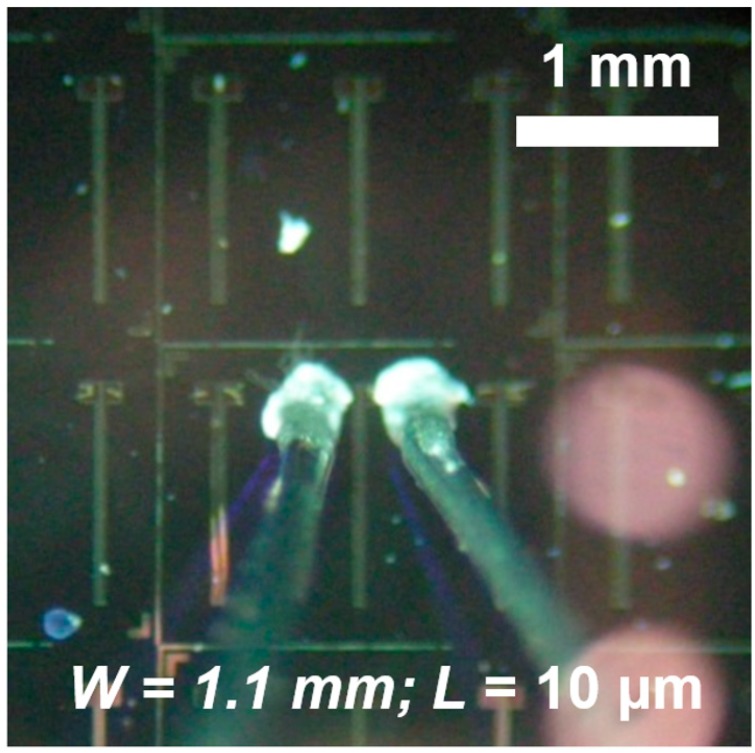
Optical micrograph of source and drain electrodes of bottom gate/bottom contact P3HT thin-film transistor (TFT) over SiO_2_ contacted through copper wires attached by conducting silver paste.

Bottom-gate bottom-contact (BGBC) TFTs were processed over highly-doped p-type Si wafers (University Wafer, MA, USA). Prior to 70-nm silicon thermal oxidation, substrates were cleaned according to the RCA procedure with a final dip in hydrofluoric acid (HF). Titanium adhesion-promoting film (10 nm) and gold source and drain electrodes (100 nm) were obtained by lift-off after electron beam physical vapor deposition (PVD). The channel width (*W*) was 1.1 mm as shown in Figure 2, while its length (*L*) varied from 4 ± 1 to 9 ± 1 µm. P3HT in 4.3 mg/mL toluene solution was spun at 1000 rpm for 30 s and then annealed according to the procedure for chemical sensors. Semiconductor coating, performed on a hexamethyldisilazane (HMDS, Sigma-Aldrich, MO, USA) vapor-treated dielectric, produced a thin-film of 29 ± 4 nm. BGBC-TFTs on silicon wafers were processed according to previous works from our group on this device structure [23], while polymeric semiconductor deposition was performed in agreement with Scarpa *et al.* [24] to produce approximately 10^−2^ cm^2^/Vs effective charge carrier mobility (μ) (see Figure S1 in the Supplementary). Atomic force microscope Nanoscope 3a Multimode 3 (Veeco/Bruker, NY, USA) operated according to Lobo *et al.* [25] and an Alpha step 100 profilometer (Veeco, NY, USA) were employed to extract thin-film thickness.

### 2.2. Gaseous Samples

Analytes injected in the chamber were ammonium hydroxide (NH_3_ (aq), *d*_v_ = 0.90 g/cm^3^, *Mw* = 35.5 g/mol, 28–30 wt ratio) 0.10–0.65 μL (67–428 ppm), ultrapure water (Millie-Q, 18.2 MΩ.cm) 0.10–1 μL (249–2486 ppm), methanol (H_3_COH, *d*_v_ = 0.79 g/cm^3^, *Mw* = 32.0 g/mol) 0.40–12 μL (443–13,284 ppm), acetone ((CH_3_)_2_CO, *d*_v_ = 0.79 g/cm^3^, *Mw* = 58.1 g/mol) 0.40–12 μL (244–7332 ppm) and chloroform (CHCl_3_, *d*_v_ = 1.48 g/cm^3^, *Mw* = 119.4 g/mol) 0.40–12 μL (222–6660 ppm). Investigation of water vapor and alcohol were motivated by their role as interferents in medical diagnosis [1,2]. All analytes, but water, were Sigma-Aldrich (MO, USA) ACS reagents. The following hypotheses were considered to calculate the analyte concentration in ppm: (1) the liquid analyte fully evaporates and diffuses through the whole chamber; and (2) 1 mole of gaseous analyte takes 22.4 L (molar volume of an ideal gas). Therefore, the concentration in ppm could be calculated according to Equation (1), where *d*_v_ is liquid analyte mass density, *V* its injected volume, *Mw* its molar mass and *V*_chamber_ the chamber volume (0.5 L). The ratio *d*_v_*V*/*Mw* corresponds to the number of moles of analyte injected from the microsyringe of 0.050-µL volume precision. 

(1) $$c(ppm)=\left(\frac{d_{v}V}{MwV_{chamber}}\right)\left(22.4\frac{L}{mol}\right)10^{6}$$

Sensitivity to water vapor was investigated due to its release from the decomposition reaction of ammonium hydroxide (NH_4_^+^ + OH^−^ ↔ NH_3_ + H_2_O) [26], and that is why the injection volumes of both analytes were similar. The concentration calculation for ammonia took into account its mass fraction in water solution, as well as the previous reaction.

### 2.3. Electronic Nose System and Sample Measurement

**Figure 3:**
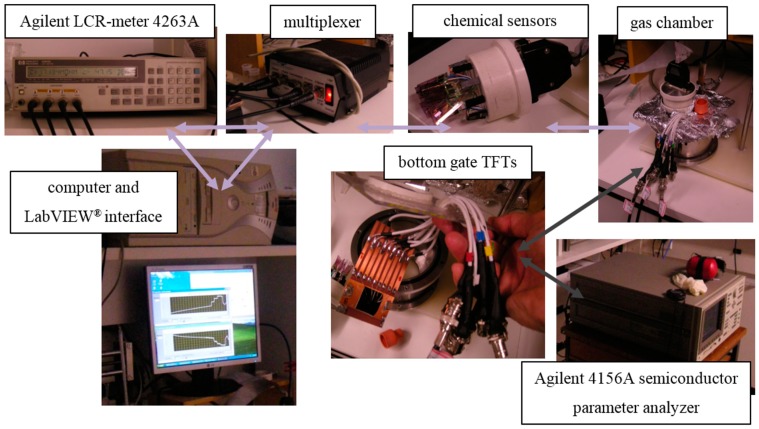
Gas detection system for chemical sensors and P3HT TFTs.

The experimental setup for sensor electrical characterization is shown in Figure 3. While chemical sensors were interrogated with a time multiplexed LCR-meter 4263 A (Agilent, CA, USA) connected to a desktop PC according to the e-tongue set-up by Braga *et al.* [22], TFTs were characterized through a 4156 A semiconductor parameter analyzer (Agilent, CA, USA). The chamber has drilled holes for positioning chemical sensors, passing electric cables to drive OTFTs, placing a septum for liquid analyte injection by a 5-µL Hamilton 7105 KH syringe of 0.1 μL/division and a gas outlet. All sensing experiments were repeated at least five times for each analyte for 2 h. pre-stabilization and a 15-min purge at 10^−2^ mbar, while keeping P3HT protected from environmental light to prevent semiconducting polymer photobleaching in the presence of oxygen and moisture [27].

### 2.4. Data Analysis

Chemical sensor resistance (*R*) and capacitance (*C*) were calculated from the *RC* parallel model obtained from the LCR-meter. On the other hand, OTFTs were monitored by the effective charge carrier mobility (µ_FET_), threshold voltage (*V*_T_) and drain current at *V*_DS_ = −1 V and *V*_GS_ = 0 V (*I*_D_). As the gate voltage sweep was performed in a short interval (±1 V) with a conducting channel already established, the current modulation (*I*_ON/OFF_) and off current (*I*_OFF_) were not calculated. All values were normalized with respect to the first measurement prior to analyte injection (atmospheric air, 1 atm, 24 °C, 60%–70% relative humidity). All electrical parameter collected data were explored under principal component analysis (PCA) [22]. Processed graphs are a biplot, which displays scores from analyte samples and loadings from sensor electrical parameters. The sensitivity of sensor electrical parameters was defined herein as their percent variation with respect to the first measurement for a 1-ppm increase in analyte concentration. The device sensitivity to ammonia was corrected by taking into account the presence of water from the ammonium hydroxide decomposition reaction. Therefore, the sensitivity of any electrical parameter (*x*) to ammonia was calculated according to Equation (2), where *k* = 2.3 ± 0.1 is the ratio between the concentration (*c*) of H_2_O and NH_3_ inside the chamber (further information is provided in Equations (1)–(9) in the Supplementary).

(2) $$\frac{\Delta x}{x_{0}}\left(NH_{3}\right)\approx \frac{\Delta x}{x_{0}}\left(NH_{4}OH\right)-k\frac{\Delta x}{x_{0}}\left(H_{2}O\right)$$

## 3. Results

**Figure 4:**
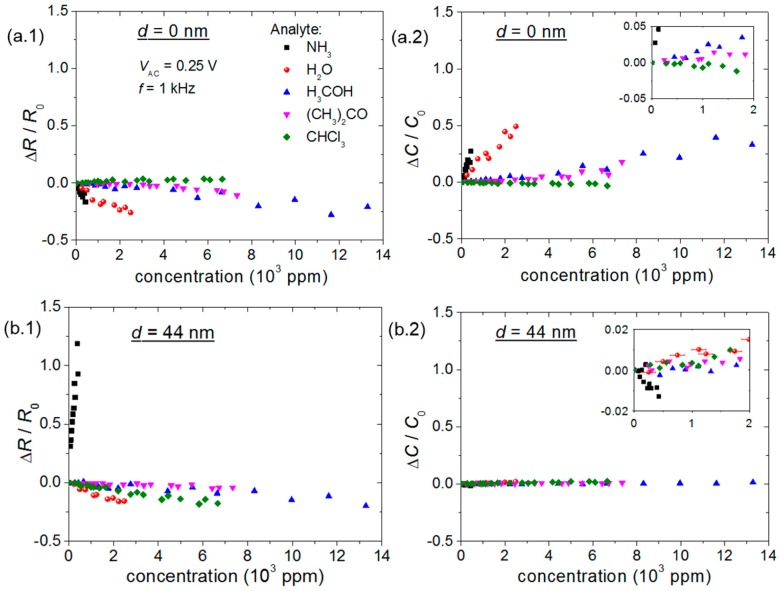

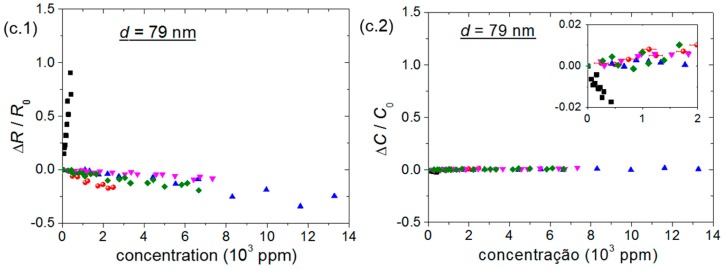
Chemical sensor electrical parameter response to gaseous analytes: (1) *R* and (2) C for *d* equal to (**a**) 0, (**b**) 44 and (**c**) 79 nm. All variations are normalized with respect to the first measurement. Except for water vapor assays, error bars are roughly the same size as the symbols.

Chemical sensor resistance (*R*) and capacitance (*C*) as a function of analyte concentration for different polymer thin-film thicknesses (*d*) are shown in Figure 4 (see Figure S4 in the Supplementary for sensor response in time). 

Results from bare sensors in Figure 4a demonstrate high sensitivity to ammonia and water vapor, associated with a negligible response to chloroform. A general trend of resistance decrease and capacitance increase is present in these devices. However, the P3HT thin-film-based devices in Figure 4b,c showed lower sensitivity to methanol and acetone than bare sensors, alongside an improved response to ammonia and chloroform. In this case, the injection of ammonium hydroxide produces the opposite effect, as resistance increases and capacitance decreases. Additionally, *R* variation predominates over *C*, as while the former varies by more than 100%, the latter changes by less than 5% at approximately 430 ppm of ammonia. Note that *R* and *C* shifts always have opposite signs for these devices, independently of *d*.

**Figure 5:**
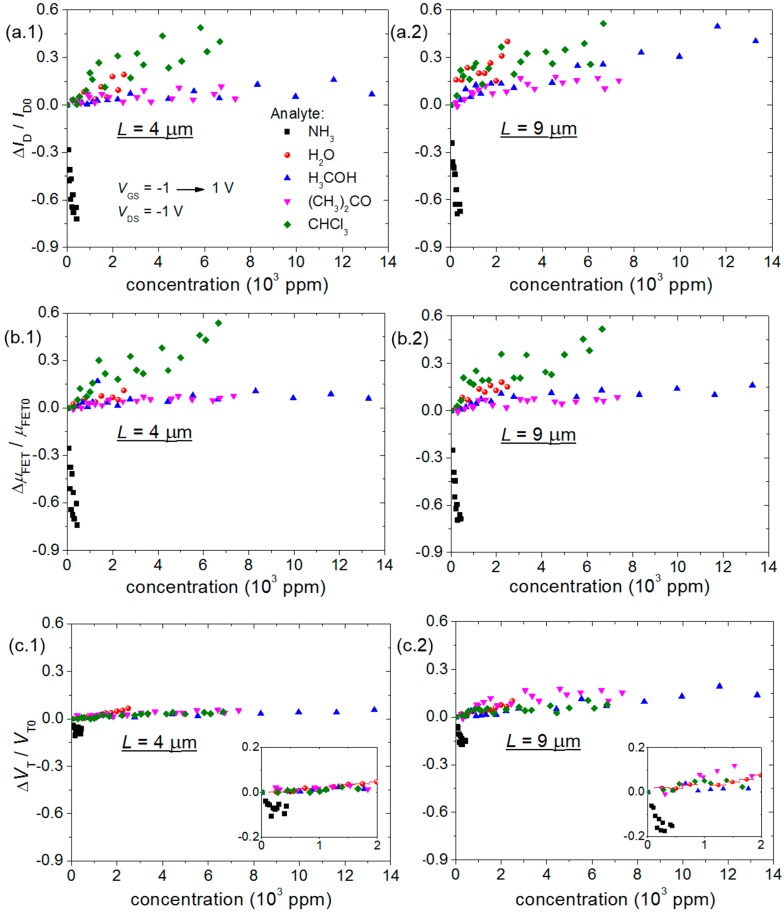
Transistor electrical parameter response to gaseous analytes: (**a**) *I*_D_, (**b**) *μ*_FET_ and (**c**) *V*_T_ for *L* equal to (1) 4 and (2) 9 μm. All variations are normalized with respect to the first measurement. Except for water vapor assays, error bars are roughly the same size as the symbols.

The P3HT transistor drain current (*I*_D_), effective charge carrier mobility (*µ*_FET_) and threshold voltage (*V*_T_) response to gaseous analytes are shown in Figure 5. The *I*_D_ and *μ*_FET_ behavior in Figure 5a,b is in agreement with *R* of P3HT chemical sensors in Figure 4b,c. Hence, a P3HT thin-film resistance change is expected to produce the opposite effect in TFT current and mobility. In another manner, *V*_T_ increases with respect to an analyte increase in concentration, except for ammonia. The response time (*t*_r_) average value for all sensors remained in an interval from 50 to 300 s (see Table S1 in the Supplementary). Sensor response depended on analyte boiling point (*bp*), as water vapor provided the longest time interval; while ammonia the shortest.

The sensitivity of chemical sensors and transistors to gaseous analytes shown in Table 1 was calculated from the slope of the linear portion of the curve for each analyte from 0 to 2000 ppm in Figure 4 and Figure 5, respectively. It is remarkable that while a linear response was already observed at the lower limit for OTFTs, it was barely noticed at the upper limit for chemical sensors. The following statements can then be settled from these results:

(i) The *R* and *C* changes of chemical sensors have opposite signs;
(ii) The highest Δ*C*/*C*_0_ of chemical sensors occurs for the bare sensor;
(iii) *I*_D_ and *μ*_FET_ variations have the opposite sign of a change in *R*;
(iv) Differently from other gaseous analytes investigated herein, an increase in ammonia concentration increases *R* of P3HT chemical sensors and decreases *I*_D_, *μ*_FET_ and *V*_T_ of transistors.

**Table 1:** Sensitivity (10^−4^ %/ppm) of chemical sensors and transistors.

Analyte	Chemical Sensors			P3HT TFTs			
	*d* (nm)	$\frac{\Delta R}{R_{0}}$	$\frac{\Delta C}{C_{0}}$	*L* (μm)	$\frac{\Delta I_{D}}{I_{D0}}$	$\frac{\Delta \mu_{FET}}{\mu_{FET0}}$	$\frac{\Delta V_{T}}{V_{T0}}$
NH_3_ *	0	−104 ± 52	150 ± 68				
	44 ± 3	2480 ± 240	−45.8 ± 7.5	4 ± 1	−2260 ± 60	−2190 ± 40	−303 ± 24
	79 ± 4	2080 ± 200	−53.1 ± 6.8	9 ± 1	−2310 ± 150	−2270 ± 80	−555 ± 35
H_2_O	0	−95.1 ± 11.6	187 ± 14				
	44 ± 3	−64.9 ± 5.7	7.1 ± 1.3	4 ± 1	62.9 ± 8.1	35.7 ± 4.2	24.0 ± 0.9
	79 ± 4	−69.4 ± 7.0	4.7 ± 1.3	9 ± 1	145 ± 17	76.9 ± 5.8	35.0 ± 1.9
H_3_COH	0	−18.5 ± 1.8	27.9 ± 2.1				
	44 ± 3	−12.1 ± 1.4	0.9 ± 0.1	4 ± 1	17.1 ± 1.7	48.3 ± 18.3	12.1 ± 1.2
	79 ± 4	−22.9 ± 2.3	0.8 ± 0.2	9 ± 1	79.4 ± 10.9	44.2 ± 3.3	16.9 ± 1.8
(CH_3_)_2_CO	0	−11.7 ± 1.1	17.1 ± 2.1				
	44 ± 3	−5.2 ± 0.7	1.0 ± 0.2	4 ± 1	36.8 ± 7.1	22.0 ± 3.6	21.1 ± 2.8
	79 ± 4	−10.8 ± 0.8	2.0 ± 0.2	9 ± 1	75.0 ± 5.5	38.7 ± 5.8	41.8 ± 3.8
CHCl_3_	0	4.7 ± 0.8	−2.9 ± 0.5				
	44 ± 3	−27.0 ± 1.5	3.4 ± 0.3	4 ± 1	143 ± 21	164 ± 26	10.8 ± 1.9
	79 ± 4	−24.7 ± 2.2	1.6 ± 0.4	9 ± 1	169 ± 22	197 ± 26	43.0 ± 4.5

* Ammonia sensitivity was calculated according to Equation (2).

**Figure 6:**
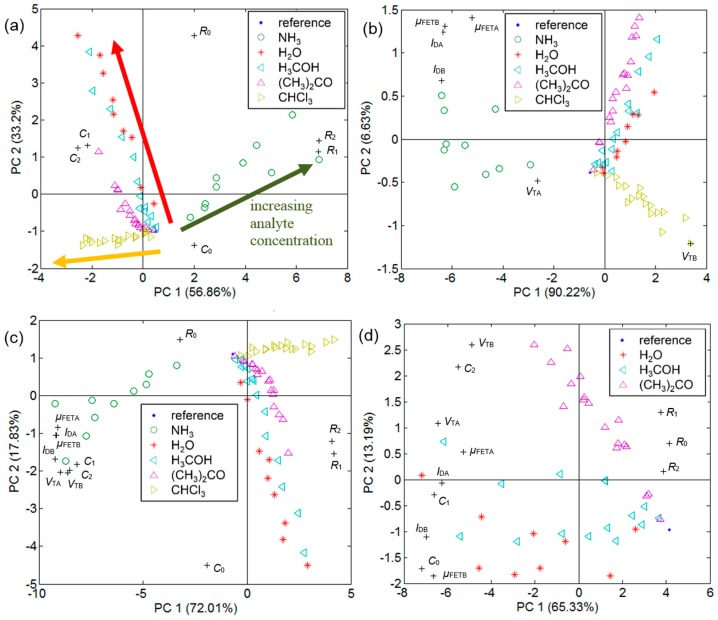
PCA biplot graph from sensor electrical parameters: (**a**) *R* and *C* for all analytes; (**b**) *I*_D_, *μ*_FET_ and *V*_T_ for all analytes; (**c**) *R*, *C*, *I*_D_, *μ*_FET_ and *V*_T_ for all analytes; (**d**) *R*, *C*, *I*_D_, *μ*_FET_ and *V*_T_ for water vapor, methanol and acetone. Legend: “0” for bare sensor, “1” and “2” for P3HT chemical sensors (*d* equal to 44 and 79 nm, respectively), “A” and “B” for P3HT TFTs (*L* equal to 4 and 9 μm, respectively).

The PCA biplot graph from *R* and *C* data of the chemical sensors demonstrates the formation of the following groups indicated by arrows in Figure 6a: ammonia, chloroform and a set defined by water vapor, methanol and acetone. Nevertheless, due to the existence of a significant dead zone in sensor response, this chemical sensor-based e-nose tends to agglomerate at least three data points from the sensor response to methanol, acetone and chloroform below 2000 ppm (see Figures S6 and S7 in the Supplementary). Notwithstanding the saturation in the TFT-based sensor response in the concentration range addressed here, PCA from their electrical parameter data produced two effects in Figure 6b:

(i) Further distinction of acetone from water vapor and methanol;
(ii) Increased distance among the lowest concentration points of chloroform, methanol and acetone.

The elimination of *V*_T_ reduced the first effect, which supports the importance of transistor behavior on acetone discrimination (see Figure S8 in the Supplementary). All sensors put together confirmed the second effect in the TFT-based e-nose, as the first principal component (PC) interval width increased from [−4, 8] in Figure 6a to [−10, 5] in Figure 6c. It is even possible to distinguish acetone from methanol and water in Figure 6d, but no tridimensional PCA plot allowed the discrimination of all analytes in the concentration range herein disclosed (see Figure S9 in the Supplementary). Variable loadings indicated as crosses (+) show that *R* and *C* take opposite sides in the PCA graph, as well as *R* and the group formed by *I*_D_, µ_FET_ and *V*_T_. The chemical sensor electrical parameters were separated in Figure 6a in the following ways:

(i) *R*_0_ from *C*_0_ along PC 2 for bare sensors and {*R*_1_, *R*_2_} from {*C*_1_, *C*_2_} along PC 1 for P3HT chemical sensors;
(ii) {*R*_0_, *C*_0_} from {*R*_1_, *R*_2_} and {*C*_1_, *C*_2_} along PC 1.

After adding the TFT electrical parameters in Figure 6c, these trends were maintained, except for *V*_T_, *I*_D_ and *μ*_FET_, which approached variables {*C*_1_, *C*_2_}. This later group could be further separated along PC 2 in two subsets, *i.e.*, {*C*_1_, *C*_2_, *V*_TA_, *V*_TB_} and {*I*_DA_, *I*_DB_, *μ*_FETA_, *μ*_FETB_}.

## 4. Discussion

### 4.1. P3HT Role in Chemical Sensors

The behavior of P3HT chemical sensors from the calculated sensitivity in Table 1 can be summarized by the following statements:

(i) The main sensing mechanism is through thin-film resistivity changes, as |Δ*R*/*R*_0_| ≫ |Δ*C*/*C*_0_|;
(ii) The highest sensitivity is in response to ammonia, the only analyte responsible for increasing *R*;
(iii) All analytes can be quantified if injected separately in the concentration range of the herein reported experiments.

P3HT films have crystalline and amorphous phases, which depend on the processing parameters, such as solvent and temperature, as well as polymer regioregularity, molecular weight and polydispersity [28,29]. The presence of a crystalline phase characterized by a lamellar structure where the planar backbone thiophene rings of the molecules are stacked on top of each other increases the charge carrier mobility in the film. Therefore, the predominance of Δ*R*/*R*_0_ changes can be expected and agrees well with previously published works on P3HT chemical sensors [30,31,32]. It is worth noticing that charge mobility is faster in the direction of conjugation, but suffers a reduction after interchain transport [28,29]. Thus, a gaseous analyte may induce changes on the crystal lattice parameters and, consequently, affect interchain charge hopping [31,32,33]. Nevertheless, as will be discussed in Section 4.2, the analyte can also alter resistance by affecting charge trapping (*i.e.*, creation, emptying or filling of localized states in energy levels close to the charge transport) [12,13] and even semiconductor doping [13].

The absence of a polymeric film in a bare sensor completely alters the sensor response. In this case, |Δ*C*/*C*_0_| ≫ |Δ*R*/*R*_0_|, the highest sensitivities are observed in response to ammonia, but also to water, while sensitivity to chloroform becomes negligible. A direct relation between Δ*C*/*C*_0_ in absolute values and the molecule electric dipole can then be established (see Table S2 in the Supplementary). The chloroform molecule, which presents the lowest electric dipole and dielectric constant values among the studied analytes [26], demands a polymeric film to enable its detection at the lowest concentration values herein. In the specific case of P3HT exposure to chloroform, it is expected that Δ*R*/*R*_0_ shifts predominate, because small concentrations of that solvent can largely change P3HT molecule diffusion, orientation, torsion and conformation in the film [33], thus improving interchain charge transport. PCA graphs from all chemical sensors in Figure 6a denote the possibility to detect ammonia, chloroform and even acetone individually, but indicate grouping of water vapor and methanol. 

Even though there are almost no reports on P3HT chemical sensors to precisely detect ammonia [30,34], previous studies have already demonstrated a polythiophene-based chemical sensor in gas sensing applications [31,32]. Li *et al.* [31] monitored conductance (*G*) alteration to detect aliphatic hydrocarbons, halogenated derivatives, aromatic compounds, alcohols, ketones and nitriles. P3HT was, in descending order, mostly sensitive to ethanol, dichloromethane and methanol through increasing *G*/*G*_0_. Additionally, their results confirmed a five-fold higher sensitivity to ethanol with respect to acetone, although these VOCs tend to gather in PCA graphs. The authors believe multiple mechanisms coexist during exposure to gaseous analytes. A chemical reaction between polymer and analyte molecules was discarded, as measurements were performed at room temperature and the sensors were reversible. Most likely, conductivity increases due to attractive electrostatic interaction among polymer molecules by induced van der Waals force after adsorption of polar VOCs. Therefore, the consequent reduced average spacing increases the available density of states for interchain polaron hopping. In flat opposition, the inverse effect is observed after exposure to nonpolar analytes due to thin-film swelling and consequent increased interchain spacing. 

A more recent study by Im *et al.* confirmed these results, but included other gaseous analytes [32]. In this case, P3HT-based sensors were, in descending order, sensitive to diisopropyl methylphosphonate (DIMP), ethanol, methanol, toluene, chloroform and hexane. PCA bidimensional graphs discriminated methanol and chloroform in agreement with our results. While the device average response time was approximately 105 s, sensor reset could take even 14 min. Similar to our results (see Figures S3 and S5 in the Supplementary), the authors limited sensing experiments to a one-month period, as O_2_ presence is believed to create instabilities in the sensor baseline, as well as prolonged exposure to high concentration of some analytes. Nevertheless, the already demonstrated alcohol detection by P3HT-based sensors finds application in other areas as a control tool for investigating gasoline adulteration at gas stations [35] and evidencing alcohol consumption in order to prevent car accidents. Furthermore, ammonia, which will be discussed in the following subsection, monitored at the concentration levels covered in this work could assist in preventing respiratory diseases and secondary infections in chicken farms (see Table S3 in Supplementary).

### 4.2. E-Nose Performance Improvement with TFTs

P3HT-based transistor results corroborate the hypothesis that the main sensing mechanism is through thin-film resistivity changes, as *I*_D_ and *µ*_FET_ vary according to *R* from chemical sensors. Even though *V*_T_/*V*_T0_ shifts less than *I*_D_/*I*_D0_ and *µ*_FET_/*µ*_FET0_ in Figure 5 in response to all analytes, the threshold voltage brings additional information to the discrimination among water vapor, methanol and acetone. Furthermore, while the chemical sensor response to methanol, acetone and chloroform clearly presents a dead zone, TFTs tend to already saturate at the lowest concentration values herein. Therefore, a hybrid e-nose makes possible not only the discrimination over a wider concentration range, but also the distinction among all studied analytes.

Contrasting with the previously discussed literature on chemical sensors, the electrical behavior of P3HT TFTs in the presence of ammonia has been widely reported [11,12,13]. Assadi *et al.* [11] monitored *μ*_FET_ and |*I*_D_| dependence on ammonia concentration for bottom gate/bottom contact P3HT TFTs on n^+^-Si/SiO_2_ (300 nm). The observed decrease after exposure to gaseous analyte was partially reversible, provided that exposure time was no longer than 20 min. Jeong *et al.* [12] included also threshold voltage shifts (Δ*V*_T_) for P3HT TFTs on p^+^-Si/SiO_2_ (100 nm) to achieve a detection limit of 10 ppm, a response time of 120–180 s and a reset time of approximately 300 s after 200 s of exposure. A Δ*V*_T_ of −13 V and *µ*_FET_/*µ*_FET0_ equal to 0.6 at 100 ppm was justified by the interaction of ammonia dipolar molecules adsorbed in the bulk or grain boundaries of the active layer in the conducting channel. Opposite of other gaseous analytes herein, these molecules are believed to behave as acceptor-like deep traps to electric charges in transport at the dielectric/semiconductor interface. Therefore, Δ*V*_T_ < 0 is likely related to changes in work function at the latter interface induced by polar molecule adsorption. 

Tiwari *et al.* [13] confirmed these observations and further reduced the detection limit until 0.1 ppm for P3HT TFTs on n^+^-Si/SiO_2_ (300 nm). Additionally, the authors observed that reset time decreases from 250 to 25 s by decreasing the concentration from 25 to 0.1 ppm. A Δ*V*_T_ of −39 V and *μ*_FET_/*μ*_FET0_ equal to 0.40 at 25 ppm was once again justified by charge transport alteration in P3HT thin-film due to charge-dipole interactions. Therefore, ammonia diffusion and adsorption in the active layer produce two correlated effects: a negative *V*_T_ shift and *μ*_FET_ decrease due to dipolar trapping. Another interpretation lies in thin-film dedoping by compensation of an ambient oxidant in P3HT. Polymer doping is mainly related to polymer synthesis, device processing or exposure to oxygen and moisture. The authors believe the presence of an oxidant atmosphere can be responsible for even higher |Δ*V*_T_|. Therefore, the resultant *I*_D_ sensitivity due to combined *V*_T_ and *μ*_FET_ simultaneous alteration has probably pushed the limit of detection of this hybrid e-nose down to units of ppm.

The possibility of employing TFTs to detect analytes at the lowest concentration values herein becomes clear after normalizing the results in Table 1 by electrode geometry (*W*/*L*). If *W* = 502 mm and *L* = 10 μm for chemical sensors and *W* = 1.1 mm and *L* = 4 or 9 μm for TFTs, then the updated device sensitivities in Table 2 can differ by three orders of magnitude. For instance, Δ*I*_D_/*I*_D0_ for *L* = 9 μm is 380-, 910-, 2700-, 5900- and 2600-times Δ*R*/*R*_0_ for *d* = 44 nm in response to ammonia, water vapor, methanol, acetone and chloroform, respectively. It becomes clear that the increased discrimination observed below 2000 ppm of the last three gaseous analytes came after including the TFT parameter dataset in the analysis. 

**Table 2:** Sensitivity (10^−6^ %/ppm) of P3HT chemical sensors and TFTs from Table 1 normalized by electrode geometry.

Analyte	P3HT Chemical Sensor		P3HT TFT			
	*d* (nm)	$\frac{\Delta R}{R_{0}}$	*L* (μm)	$\frac{\Delta I_{D}}{I_{D0}}$	$\frac{\Delta \mu_{FET}}{\mu_{FET0}}$	$\frac{\Delta V_{T}}{V_{T0}}$
NH_3_ *	44 ± 3	4.94 ± 0.48	4 ± 1	−824 ± 23	−797 ± 13	−110 ± 9
	79 ± 4	4.14 ± 0.39	9 ± 1	−1890 ± 120	−1860 ± 60	−454 ± 29
H_2_O	44 ± 3	−0.129 ± 0.011	4 ± 1	22.9 ± 2.9	13.0 ± 1.5	8.7 ± 0.3
	79 ± 4	−0.138 ± 0.014	9 ± 1	118 ± 14	62.9 ± 4.7	28.6 ± 1.6
H_3_COH	44 ± 3	−0.024 ± 0.003	4 ± 1	6.2 ± 0.6	17.6 ± 6.7	4.4 ± 0.4
	79 ± 4	−0.046 ± 0.005	9 ± 1	65.0 ± 8.9	36.2 ± 2.7	13.8 ± 1.5
(CH_3_)_2_CO	44 ± 3	−0.010 ± 0.001	4 ± 1	13.4 ± 2.6	8.0 ± 1.3	7.7 ± 1.0
	79 ± 4	−0.022 ± 0.002	9 ± 1	61.4 ± 4.5	31.7 ± 4.7	34.2 ± 3.1
CHCl_3_	44 ± 3	−0.054 ± 0.003	4 ± 1	52.1 ± 7.7	59.6 ± 9.5	3.9 ± 0.7
	79 ± 4	−0.049 ± 0.004	9 ± 1	138 ± 18	161 ± 21	35.2 ± 3.7

* Ammonia sensitivity was calculated according to Equation (2).

Chemical sensors from 44 or 79 nm-thick P3HT film provide similar normalized sensitivities in Table 2. The almost 45% film thinning increased Δ*R*/*R*_0_ to ammonia by 19%, while Δ*I*_D_/*I*_D0_ represents a 20,000%–46,000% improvement. Such signal amplification by two to even three orders of magnitude can only be related to the TFT gate electrode and, consequently, the combined effect on *I*_D_ of *µ*_FET_ and *V*_T_. The last parameter depends, among others, on fixed and mobile charges in the inorganic dielectrics, interface traps for charge transport in the dielectric/semiconductor interface and the Fermi level difference between the gate electrode and semiconductor [36]. Similarly to the ammonia effects on P3HT TFTs studied by Tiwari *et al.* [13], we believe the main effect on *V*_T_ after exposure to a gaseous analyte is an alteration in interface states between SiO_2_ and P3HT, as well as P3HT doping, which, in turn, affect the density of traps for charge transport and the Fermi level of the semiconductor. Therefore, the overall effect of each gas on channel resistance could be summarized as follows: (i) *µ*_FET_ and *V*_T_ decrease in a p-type semiconductor by an oxidizing gas, such as ammonia; and (ii) *µ*_FET_ and *V*_T_ increase in a p-type semiconductor by a reducing gas, such as water vapor, methanol, acetone and chloroform [37].

It is also well known that charge transport takes place in an accumulated channel thickness less than or equal to 10 nm after the application of a positive gate voltage (*V*_GS_) lower or more negative than *V*_T_ in P3HT TFTs [20,21]. As *d* = 29 nm in TFTs represents only a 63% thinning of the active layer, the transistor improved sensitivity points out that electric current flows significantly in a much smaller thickness. The P3HT thin-film thickness in TFTs agrees well with previous works [38,39] and was chosen in order to reduce the off current and improve current modulation. According to Gburek and Wagner [38], *I*_OFF_ increases by one order of magnitude by varying *d* from 216 to 21 nm. By contrast, Park *et al.* [39] showed that nanometric P3HT films (*d* ≤ 6 nm) can lead to higher disorder and lower hole mobility. Furthermore, even though a rougher film should provide a higher interaction area with gaseous analytes on the external surface and grain boundaries [12,13], analysis of AFM micrographs of P3HT thin-films (see Figure S2 in the Supplementary) shows that root-mean-square roughness (*R*q) decreases from 3.28 to 1.42 and further to 1.15 nm by reducing *d* from 79 to 44 and to 29 nm, respectively. Thus, emphasizing the role of the gate electrode in signal amplification, TFTs with the lowest *R*q values among all of the investigated devices presented the highest normalized sensitivities herein. Finally, further miniaturization will not necessarily provide a better sensing device. According to Table 2, Δ*I*_D_/*I*_D0_ for *L* = 4 μm is 0.44-, 0.19-, 0.10-, 0.22- and 0.37-times the results for 9 µm in response to ammonia, water vapor, methanol, acetone and chloroform, respectively. In this case, an eventual *I*_D_ reduction due to an *L* increase can be easily compensated by an increased sensitivity mainly to water vapor, methanol and acetone. 

Even though the injected volume of ammonium hydroxide is 1/10 of methanol, acetone and chloroform amounts, sensitivity to ammonia is one or even two orders of magnitude higher than to all the other targeted gases. In spite of this, if the evaporation time of water at room temperature from the ammonium hydroxide decomposition reaction is neglected, the response time (*t*_r_) upon exposure to ammonia is comparable to what is observed for methanol, acetone and chloroform (see Table S1 in the Supplementary). Although sensors respond 4 s after NH_3_ (aq) injection, signal stabilization takes 1–2 min upon 100–200 ppm of ammonia. High concentration values, such as 300 ppm, can also delay the reset time to longer intervals, such as 25 min (see Figure S5 in the Supplementary). According to Tiwari *et al.*’s results [13], a reduced analyte concentration (<10 ppm) could reduce *t*_r_ and even reset time to less than 30 and 100 s, respectively. The superior performance of their devices is probably related to the higher applied electric potential and lower ammonia concentration. Hence, a more aggressive *W*/*L* design, such as the interdigitated geometry of source and drain electrodes, could certainly bring the limit of detection of polymer TFTs herein below 1 ppm to identify Helicobacter pylori colonies in gastrointestinal tract [2] or even to diagnose renal [6] and hepatic diseases [7].

Chang *et al.* [9] investigated polythiophene derivative sensitivity to VOCs in TFTs over n^+^-Si/SiO_2_ (95 nm). The results from spin-coated P3HT showed high Δ*V*_T_/*V*_T0_ in response to organic acids and Δ*μ*_FET_/*μ*_FET0_ to alcohols, aldehydes and amines. In agreement with our results, the alcohol-enriched atmosphere produces positive *V*_T_ shifts. However, a direct relation between sensitivity and alkane chain length implied the lowest sensitivity to methanol. Additionally, the authors remarked that the polythiophene-based sensor response to alcohols was reversible, differently from most inorganic sensors. In addition to previously cited alcohol-related applications, increased detection of acetone should make these polymer TFTs available for non-invasive glucose concentration evaluation [3,4]. 

Although oxygen and moisture are well-known to degrade P3HT-based transistor and photovoltaic performance [27,40], these results demonstrate that both kinds of polymer-based devices, *i.e.*, chemical sensors and thin-film transistors, can simultaneously compose an e-nose with a wide concentration range (1–10,000 ppm) for applications in disease diagnosis and environment monitoring. Future studies should demonstrate sensor reusability according to the reversible behavior without exposing devices to high ammonia and chloroform concentrations. Further design research on electrode geometry, signal processing and inserting other polymeric semiconductors should improve the detection limit of acetone and ammonia to less than 1 ppm, therefore accrediting this hybrid e-nose for medical applications.

## 5. Conclusions

P3HT-based chemical sensors were sensitive to all studied gaseous analytes, *i.e.*, ammonia, water vapor, methanol, acetone and chloroform. A one order of magnitude higher sensitivity was observed in response to ammonia with respect to other analytes. The presence of a semiconducting polymer film in these devices compared to a sensor with bare electrodes affected performance as follows: (i) the sensing mechanism was altered in thin-film resistance instead of capacitance; (ii) the highest sensitivities changed from water vapor and ammonia to only the latter; (iii) chloroform, which was almost undetectable by the bare sensor, turned out to be easily discriminated on PCA graphs. The adsorption of polar VOCs, such as acetone and methanol, reduces the average spacing among polymer molecules by induced van der Waals force, therefore increasing π molecular orbital stacking and thin-film conductivity. Exposure to chloroform produces a similar behavior, as this is a common solvent for preparing P3HT solutions and has the potential to directly affect thin-film morphology. Ammonia, on the other hand, behaves as acceptor-like deep traps to electric charges in transport due to nitrogen’s high electronegativity and available lone pairs. Additionally, it may act as a dedoping agent by compensating for an ambient oxidant in P3HT, such as moisture or oxygen. 

Deposition of P3HT over p^+^-Si/SiO_2_ to produce bottom gate/bottom contact thin-film transistors improved normalized sensitivity by three orders of magnitude. Even though TFTs were prone to saturate in the concentration range herein, the limit of detection for ammonia is likely reduced to units of ppm, as well as for acetone and chloroform to tens of ppm. Simultaneous variation in threshold voltage and effective charge carrier mobility in a thin accumulated charge transport channel is believed to generate an amplified response compared to chemical sensors. The integration of both kinds of devices in an electronic nose analyzed by PCA improved both the detection range and analyte discrimination. A more aggressive *W*/*L* ratio by channel length reduction impaired sensor performance. Interdigitated electrodes should further push the detection limit to even below 1 ppm, while alternative semiconducting polymers could integrate such a hybrid e-nose to improve the discrimination of methanol from water vapor.

## Supplementary Files

Supplementary File 1
