# Supplementary material for: Enhanced Sensitivity of Gas Sensor Based on Poly(3-hexylthiophene) Thin-Film Transistors for Disease Diagnosis and Environment Monitoring
Source: Sensors (Basel). 2015 Apr 22;15(4):9592–609. doi: 10.3390/s150409592 (PMC4431216; doi:10.3390/s150409592)
Supplement: Supplementary File 1 [file sensors-15-09592-s001.pdf]

OPEN ACCESS

sensors

ISSN 1424-8220

www.mdpi.com/journal/sensors

Supplementary Information

Enhanced Sensitivity of Gas Sensor Based on Poly(3-hexylthiophene) Thin-Film Transistors for Disease Diagnosis and Environment Monitoring. *Sensors* 2015, *15*, 9592-9609

Marco R. Cavallari [1], José E. E. Izquierdo [1,2], Guilherme S. Braga [1,3], Ely A. T. Dirani [1,4], Marcelo A. Pereira-da-Silva [5,6], Estrella F. G. Rodríguez [2] and Fernando J. Fonseca [1,*]

1 Departamento de Engenharia de Sistemas Eletrônicos, Escola Politécnica da Universidade de São Paulo (EPUSP), Av. Prof. Luciano Gualberto, trav. 3, n. 158, Cidade Universitária, CEP 05508-900, São Paulo-SP, Brasil; E-Mails: rcavallari@lme.usp.br (M.R.C.); jeeizquierdo@lme.usp.br (J.E.E.I.); gbraga@lme.usp.br (G.S.B.); dirani@pucsp.br (E.A.T.D.)

2 Instituto Superior Politécnico José Antonio Echeverría (ISPJAE), Centro de Investigaciones de Microelectrónica (CIME), Antigua Carretera de Vento, km 8 1/2, Boyeros, CP 10800 La Habana, Cuba; E-Mail: estrella@electrica.cujae.edu.cu

3 EMBRAPA Instrumentação, Rua Quinze de Novembro, 1452-Centro, CEP 13560-970 São Carlos, SP, Brasil.

4 Pontifícia Universidade Católica de São Paulo (PUC-SP), Rua Marquês de Paranaguá, 111-Consolação, CEP 01303-050 São Paulo-SP, Brasil

5 Instituto de Física de São Carlos–USP, Av. Trabalhador Sãocarlense 400, CEP 13566-590 São Carlos-SP, Brasil; E-Mail: maps@ifsc.usp.br

6 Centro Universitário Central Paulista–UNICEP, Rua Miguel Petroni 5111, CEP 13563-470 São Carlos-SP, Brasil

* Author to whom correspondence should be addressed; E-Mail: fernando.fonseca@poli.usp.br; Tel.: +55-11-3091-5257; Fax: +55-11-3091-5585.

Characteristic curves of P3HT bottom-gate bottom-contact thin-film transistors (TFTs) over SiO_2 are given in Figure S1. Calculated electrical parameters from I_D *versus* V_{GS} curves in Figure S1b are μ_{FET} = (2.6 ± 0.2) × 10^{-2} cm^2/Vs, V_T = (10 ± 2) V and $I_{ON/OFF}$ = 23 ± 15. A thin-film thickness (*d*) of 29 nm was achieved by decreasing the solution concentration from 7 to 4.3 mg/mL with respect to chemical sensors, as the spinning frequency was already 3000 rpm and provided 44 nm. A thinner film is desirable in organic TFTs in order to reduce the off current, to improve current modulation and, thus, to better observe the effects of each analyte on TFTs' electrical parameters [1]. Atomic force microscopy (AFM) micrographs (processed in Gwyddion (v.2.40) free scanning probe microscopy (SPM) data analysis software) of P3HT thin-films are shown in Figure S2. Even though TFTs present the highest

sensitivities normalized by electrode geometry in this work, the thin-films employed in chemical sensors are not only thicker, but also rougher than the ones in TFTs.

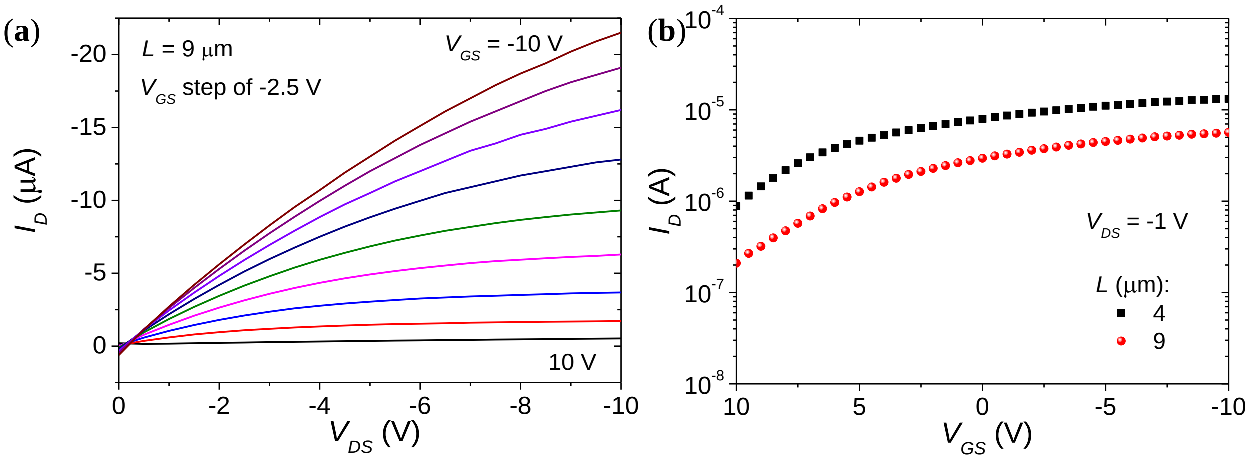

Figure S1. Characteristic curves of P3HT transistors: (**a**) I_D *versus* V_{DS} for V_{GS} from 10 to −10 V; (**b**) I_D *versus* V_{GS} for V_{DS} = −1 V.

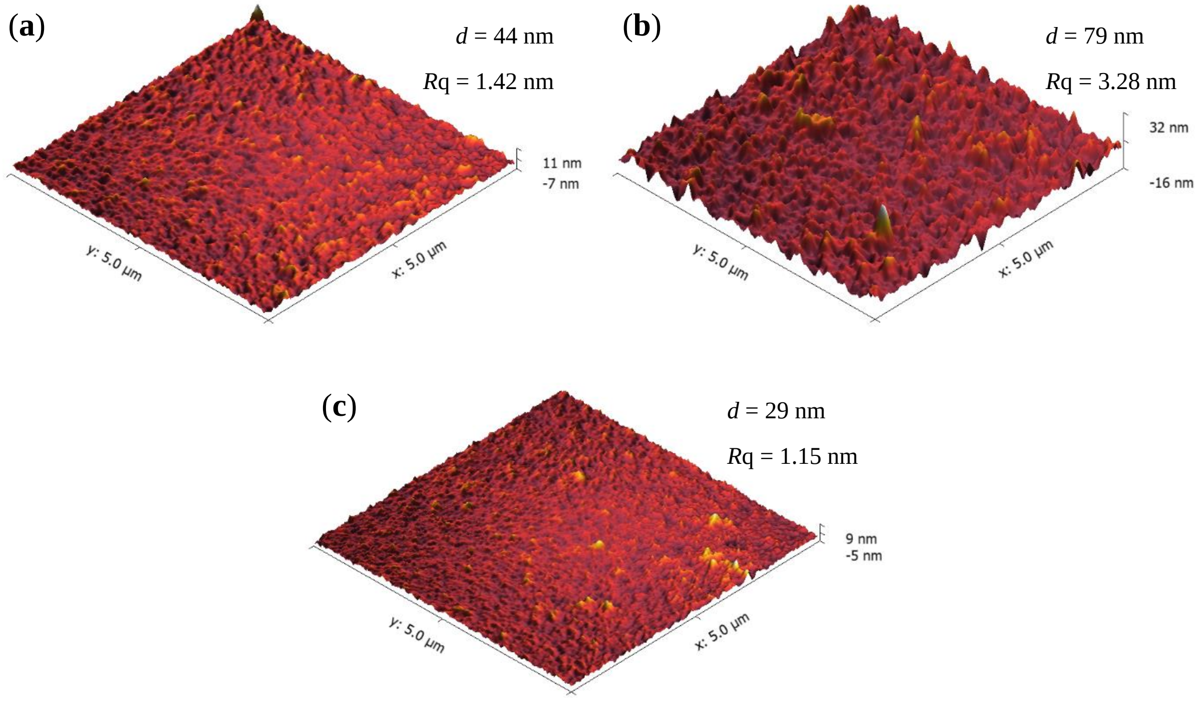

Figure S2. AFM micrographs and root-mean-square roughness (*R*q) of P3HT thin-films with thickness equal to: (**a**) 44 and (**b**) 79 nm in chemical sensors; (**c**) 29 nm in TFTs.

Device sensitivity to ammonia was corrected by taking into account the presence of water from the ammonium hydroxide decomposition reaction according to Equation (1).

$$\frac{\Delta x}{x_0}(NH_3) \approx \frac{\Delta x}{x_0}(NH_4OH) - k\frac{\Delta x}{x_0}(H_2O) \quad (1)$$

Considering the *w* ammonia weight ratio in the aqueous solution of volume $V_{liq.}$, dissolved ammonia and water number of moles (*n*) can be obtained according to Equations (2)–(6), where *d* is density, *m* is mass and *M*w is molecular weight.

- Mass calculation: $m_{NH_4OH(l)} = d_{NH_4OH(l)}V_{liq.}$

$$m_{NH_4OH(l)} = d_{NH_4OH(l)}V_{liq.} \tag{2}$$

$$m_{NH_3(l)} = wm_{NH_4OH(l)} \tag{3}$$

$$m_{H_2O(l)} = (1-w)m_{H_2O(l)} \tag{4}$$

- Number of moles calculation:

$$n_{NH_3(l)} = \frac{m_{NH_3(l)}}{Mw_{NH_3}} \tag{5}$$

$$n_{H_2O(l)} = \frac{m_{H_2O(l)}}{Mw_{H_2O}} \tag{6}$$

Additionally, considering that (1) the liquid analyte fully evaporates and diffuses through the whole volume of the chamber ($V_{chamber}$) and (2) 1 mole of gaseous analyte takes 22.4 L (molar volume of an ideal gas), the concentration (*c*) in ppm can be calculated according to Equations (7) and (8).

$$c_{NH_3(g)}(ppm) = \left(\frac{n_{NH_3(g)}}{V_{chamber}}\right)\left(22.4\frac{L}{mol}\right)10^6 \tag{7}$$

$$c_{H_2O(v)}(ppm) = \left(\frac{n_{H_2O(v)}}{V_{chamber}}\right)\left(22.4\frac{L}{mol}\right)10^6 \tag{8}$$

Finally, the ratio between the concentration of water vapor and ammonia inside the chamber, *k*, can be calculated according to Equation (9).

$$k = \frac{c_{H_2O(v)}(ppm)}{c_{NH_3(g)}(ppm)} \tag{9}$$

Sensing experiments were performed in one months' time due to persistent degradation of P3HT sensor baseline response after exposure to high concentrations of ammonia and chloroform gaseous analytes. Clear spots appeared in violet P3HT thin-film in Figure S3 as a consequence of polymer dissolution and chemical degradation in the presence of the previously cited analytes and atmospheric gases. Differently from chemical sensors, the P3HT TFT electrical parameter responses were still stable after this time period.

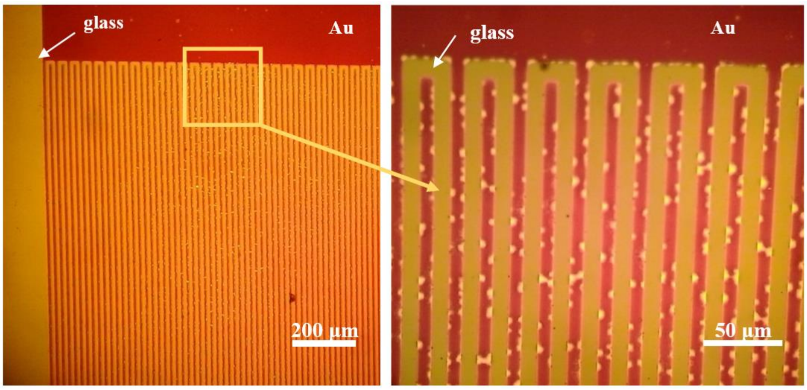

Figure S3. Optical micrographs of a P3HT chemical sensor after the experiments.

Chemical sensor resistance (R) response to gaseous analytes for different polymer thin-film thicknesses (d) are shown in Figure S4.

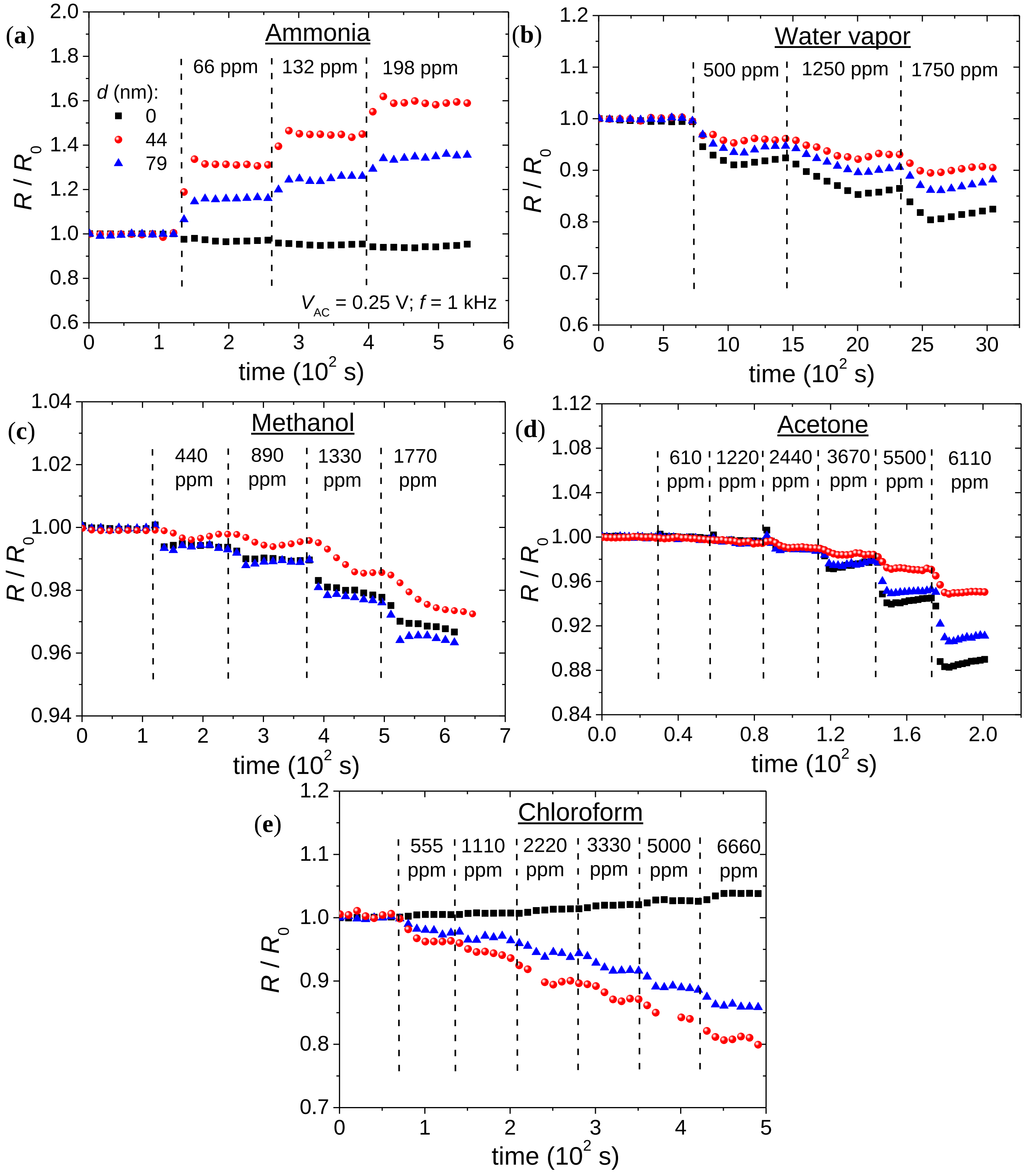

Figure S4. Chemical sensor R response to gaseous analytes: (**a**) ammonia; (**b**) water vapor; (**c**) methanol; (**d**) acetone; and (**e**) chloroform. All variations are normalized with respect to the first measurement.

The response time (t_r) average value of all sensors given in Table S1 remained within the error interval for each analyte. Higher analyte concentrations implied longer intervals. This represented an increase of 35, 56 and even 77% after triplicating the concentration values shown in Table S1 in response to acetone, methanol and chloroform, respectively.

Table S1. Response time (t_r) of chemical sensors and transistors.

**Analyte**	***c (ppm)***	***bp* (℃)**	**t_r (s)**
NH_3	99 ± 16	28.3	52 ± 4
H_2O	249 ± 62	100.0	298 ± 4
H_3COH	1100 ± 27	64.5	161 ± 4
$(CH_3)_2CO$	1200 ± 15	56.5	68 ± 4
$CHCl_3$	560 ± 14	61.0	201 ± 4

The reset time of P3HT-based sensors is also dependent on analyte concentration. Sensor response to approximately 300 ppm of ammonia is shown in Figure S5. Compared to a bare sensor, while the former has already been reset in Figure S5a, both P3HT chemical sensors and TFTs did not return to pristine values after 25 min.

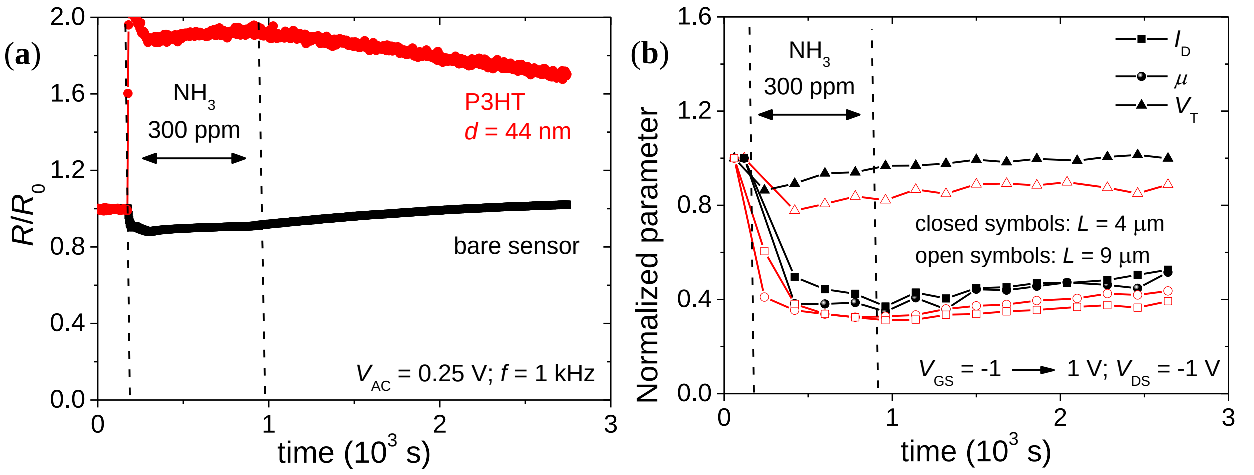

Figure S5. Response and reset times of the sensor's electrical parameters under exposure to approximately 300 ppm of ammonia: (**a**) *R* from chemical sensors; (**b**) I_D, μ and V_T of P3HT TFTs. All parameters are normalized with respect to the first measurement.

The capacitance response of bare chemical sensors is related to the analyte dielectric constant (κ) and electric dipole (μ_D) in Table S2. An insignificant response to chloroform is due to the direct relation between molecule polarity and sensor response.

Principal component analysis (PCA) from both chemical sensor and TFT electrical parameters in Figure S6 emphasizes the existence of directions through which the concentration changes. The drawn arrows depart from the three lowest analyte concentration values around the reference dot, which are represented by smaller symbols, and point to the three highest analyte concentration values, which are represent by bigger symbols.

Table S2. Capacitance sensitivity (10^{-4} %/ppm) of bare sensor against dielectric constant (κ) and electric dipole (μ_D) of studied gaseous analytes.

**Analyte**	**κ* (20 °C)**	**μ_D * (D)**	$\left\|\frac{\Delta C}{C_0}\right\|$
NH_3 *	16.61	1.4718 ± 0.0002	150 ± 68
H_2O	80.1	1.8546 ± 0.0040	187 ± 14
H_3COH	33.0	1.70 ± 0.02	27.9 ± 2.1
$(CH_3)_2CO$	21.01	2.88 ± 0.03	17.1 ± 2.1
$CHCl_3$	4.81	1.04 ± 0.02	2.9 ± 0.5

* According to [2].

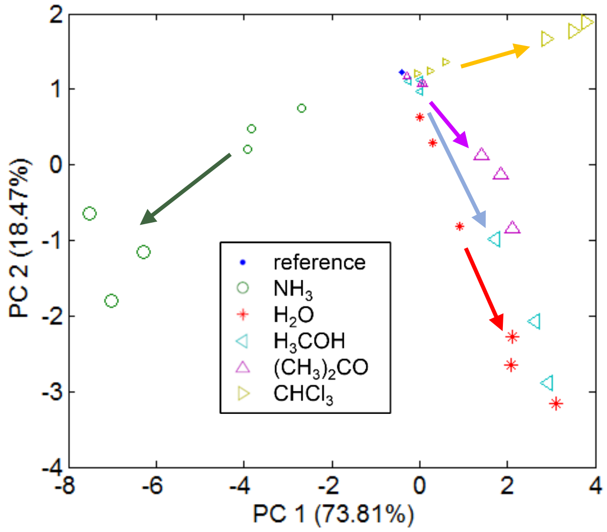

Figure S6. PCA graph from both chemical sensor (R, C) and TFT electrical parameters (I_D, μ_{FET} and V_T) at the three lowest analyte concentration values (smaller symbols) and the three highest analyte concentration values (bigger symbols). Arrows are a guide to the eye towards increasing analyte concentration.

A comparison between chemical sensors and P3HT TFT performance at low and high analyte concentrations is given in the PCA biplot graphs of Figure S7. Even though increasing concentration points towards different directions on the plane defined by the first two principal components in Figure S7a, the intersection of the ellipses drawn from the chemical sensor response to less than 2000 ppm of methanol, acetone and chloroform tended to enclose at least three data points for each of these analytes. As will be shown later, this relates to the presence of a noticeable dead zone in sensor response at the lowest concentration values herein. On the other hand, organic TFTs shown in Figure S7b increased the ellipse areas at the same concentration range. This phenomenon is observed also for ammonia at less than 200 ppm. Furthermore, acetone can be fully discriminated from other analytes below 2000 ppm only with a TFT-based e-nose.

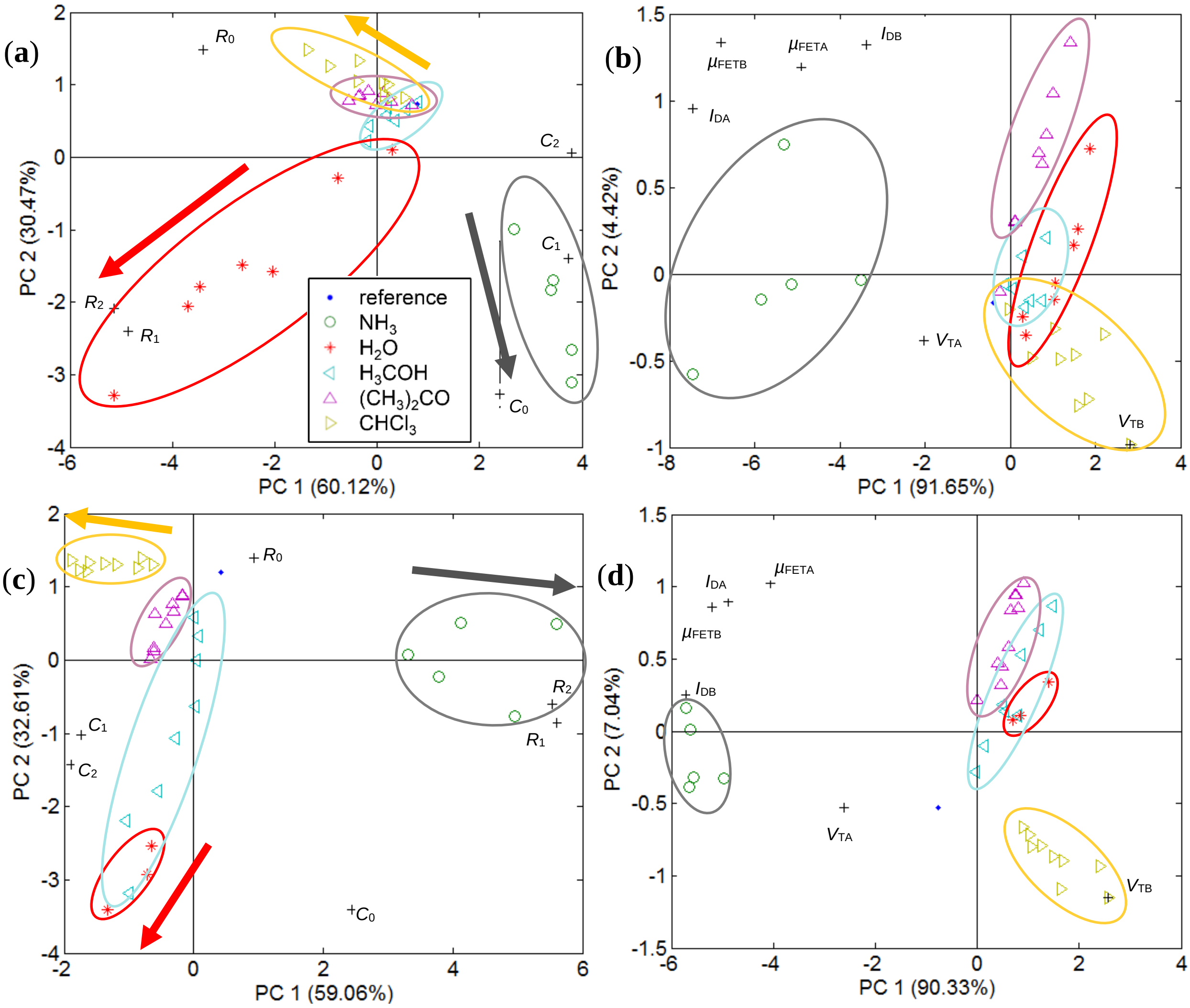

Figure S7. PCA biplot graph from sensor electrical parameters: (**a**) *R*, *C*, (**b**) I_D, μ_{FET} and V_T at a low analyte concentration (<200 ppm for ammonia and <2000 ppm for other gases); (**c**) *R*, *C*, (**d**) I_D, μ_{FET} and V_T at a high analyte concentration (>200 ppm for ammonia and >2000 ppm for other gases). Arrows are a guide to the eye towards increasing analyte concentration. Legend: "0" for bare sensor, "1" and "2" for P3HT chemical sensors (*d* is equal to 44 and 79 nm, respectively), "A" and "B" for P3HT TFTs (*L* is equal to 4 and 9 µm, respectively).

Increasing analyte concentration ameliorated acetone and chloroform discrimination by chemical sensors in Figure S7c with respect to Figure S7a. On the other hand, TFTs showed the effects of sensor response saturation in analyte concentration determination, as ellipses for all analytes, but methanol are reduced in size in Figure S7d compared to Figure S7b. Moreover, the acetone data are no longer fully isolated from the methanol ellipse. In comparison to chemical sensors at a high analyte concentration, P3HT TFTs tended to agglomerate data points for ammonia at more than 200 ppm and to overlap data for water vapor, methanol and acetone at more than 2000 ppm. The elimination of threshold voltage (V_T) data in Figure S8 completely altered the response of the P3HT TFT-based e-nose. The presence of this parameter, which has no similarity in chemical sensors, reduced the superimposition of ellipses for all analytes, except ammonia, and further stretched the ellipses due to varying analyte concentrations.

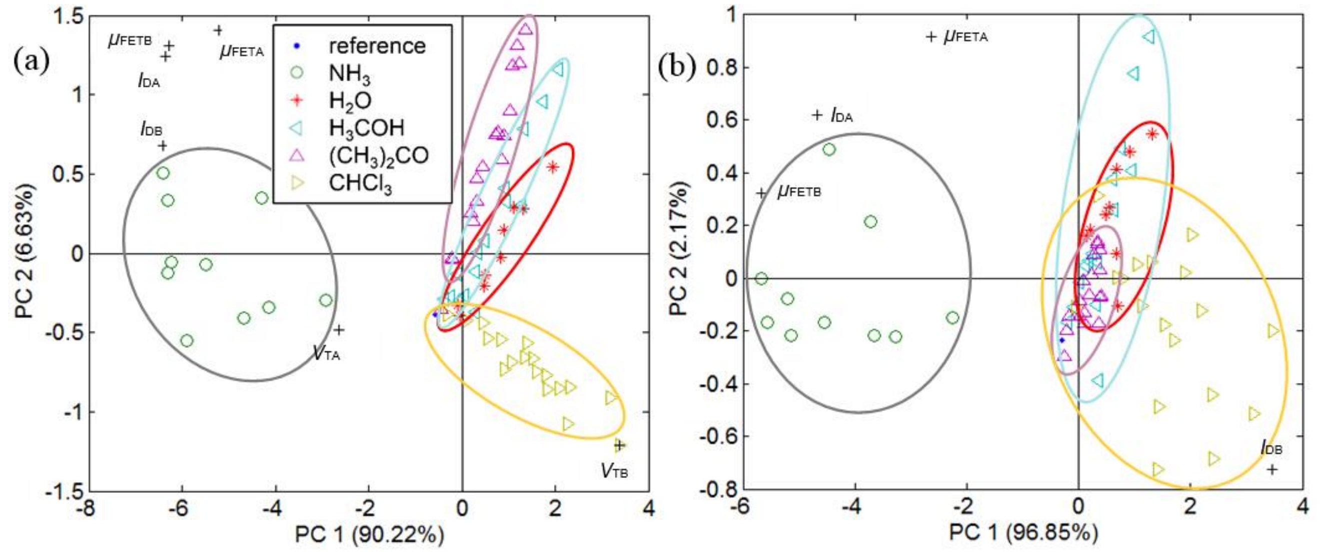

Figure S8. PCA biplot graph from P3HT TFT electrical parameters: (**a**) I_D, μ_{FET} and V_T; (**b**) I_D and μ_{FET}. Legend: "A" and "B" for L equal to 4 and 9 µm, respectively.

No herein studied e-nose was fully capable of differentiating methanol from water vapor, even after including a third principal component in the PCA plots. There was no viewing angle from which methanol could be separated from water vapor in Figure S9a, independently of the electrical parameters and devices employed to perform this statistical analysis. Nevertheless, full distinction is achieved at a definite concentration, such as at approximately 2000 ppm in Figure S9b.

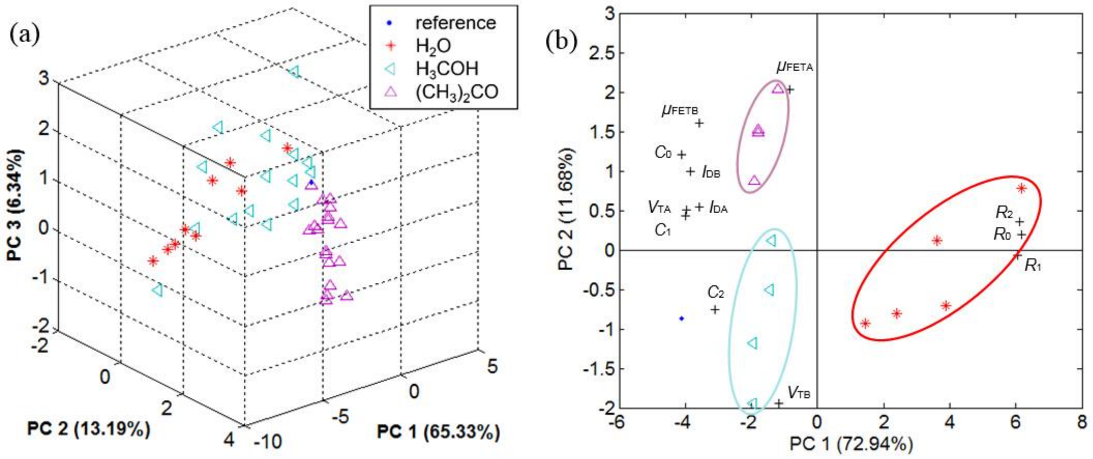

Figure S9. PCA graphs from all sensor electrical parameters in the presence of water vapor, methanol and acetone: (**a**) tridimensional plot; (**b**) biplot for $1500 < c < 2500$ ppm. Legend: "0" for bare sensor, "1" and "2" for P3HT chemical sensors (d is equal to 44 and 79 nm, respectively), "A" and "B" for P3HT TFTs (L is equal to 4 and 9 µm, respectively).

The limit of detection (LOD) of chemical sensors calculated according to [3] from the data plotted in Figures 3 and 4 of the manuscript is provided in Table S3. It demonstrates that an e-nose based on chemical sensors can monitor 25 ppm of ammonia for chicken farms, but requires P3HT TFTs for the detection of acetone and methanol below 100 ppm or even ammonia below 5 ppm for disease diagnosis.

All TFTs are already approaching saturation in the concentration range addressed in this work and, therefore, provide a negative LOD.

Table S3. Limit of detection * (LOD in ppm) of chemical sensors.

Analyte	LOD of Bare Sensors (d = 0 nm)	LOD of P3HT Sensors (d = 44 nm)	LOD of P3HT Sensors (d =79 nm)
NH_3	12 ± 4	<16 **	16 ± 8
H_2O	26 ± 7	46 ± 12	140 ± 20
H_3COH	370 ± 40	570 ± 40	780 ± 70
$(CH_3)_2CO$	490 ± 60	440 ± 160	190 ± 70
$CHCl_3$	190 ± 40	110 ± 30	69 ± 34

* According to [3], LOD is the concentration at which the extrapolated linear portion of the calibration graph intersects the baseline of the calibration curve; ** Sensor already reaching saturation.

References

1. Gburek, B.; Wagner, V. Influence of the semiconductor thickness on the charge carrier mobility in P3HT organic field-effect transistors in top-gate architecture on flexible substrates. *Org. Electron.* **2010**, *11*, 814–819.
2. Lide, D.R. *CRC Handbook of Chemistry and Physics*, 86th ed.; Publisher: CRC Press: Boca Raton, FL, USA, 2005.
3. Grate, J.W.; Abraham, M.H. Solubility interaction and design of chemically selective sorbent coatings for chemical sensor and arrays. *Sens. Actuators B Chem.* **1991**, *3*, 85–111.

© 2015 by the authors; licensee MDPI, Basel, Switzerland. This article is an open access article distributed under the terms and conditions of the Creative Commons Attribution license (http://creativecommons.org/licenses/by/4.0/).
